# Micro-computed tomography scan and virtual histological slide data for the land planarian *Obama otavioi* (Platyhelminthes)

**DOI:** 10.1186/s13742-016-0119-4

**Published:** 2016-03-16

**Authors:** Fernando Carbayo, Jennifer Winifred Lenihan

**Affiliations:** Laboratório de Ecologia e Evolução, Escola de Artes, Ciências e Humanidades, Universidade de São Paulo - USP, Av. Arlindo Bettio, 1000, CEP 03828-000 São Paulo, SP Brazil; Museum of Comparative Zoology, Department of Invertebrate Zoology, Harvard University, Cambridge, Massachusetts USA

**Keywords:** μCT, MicroCT, Slide digitization, Anatomy, Histology, Taxonomy, Morphology, Geoplanidae, Tricladida, Soft-tissue

## Abstract

**Background:**

We investigated whether images obtained through X-ray micro-computed tomography (μCT) can be used in conjunction with traditional methods for morphological studies of soft-bodied land planarians. μCT is non-invasive and provides true-to-scale three-dimensional imagery at high resolution. We compared μCT-based images of a recently described land planarian species of *Obama otavioi* (Platyhelminthes) with those obtained from light microphotography of histological sections, most of which were also digitized at high magnification.

**Findings:**

The specimens studied were collected in 2012. Subsequent μCT-based images of the stained body of a paratype show nearly all morphological features provided by traditional histology, with the exception of particularly minute structures, smaller than 5 μm, such as the sensory pits and single muscle fibers, which are best visible on traditional histological sections. Because the technique is non-destructive, the scanned specimen is preserved without damage. The raw and derivative μCT data and virtual histological sections are freely available in GigaDB.

**Conclusions:**

The μCT datasets of these stained soft-bodied organisms reveal images of external and internal structures that support previous taxonomic studies. This technique can be particularly important for non-destructively revealing internal details of whole museum specimens at a faster rate than histology alone. High-resolution virtual histological slides also allow further searches for new, previously unstudied morphological features. The use of X-ray equipment with higher resolution can enable smaller sensory organ and muscle fiber details to be seen. The image sets, μCT-based images and digitized histological slides can be disseminated without the constraints of specimen loans.

## Data description

### Purpose of data acquisition

Unequivocal identification and comprehensive description of a diversity of animal taxa depend on destructive techniques, such as traditional histological sections. This technique is laborious and may result in patchy and distorted sections [1, 2]. Current taxonomic studies of land planarians (Platyhelminthes, Tricladida) require histological sectioning. Furthermore, some land planarian species of the genus *Obama* (Geoplaninae, a group of exclusively neotropical land planarian species) are morphologically very similar to their relatives and taxonomically relevant organs, such as the copulatory apparatuses, are internally asymmetrical so histological sections often hinder reconstruction of these structures [3].

Many species of Geoplaninae are poorly represented in museum collections. Destructive techniques provide morphological information along with a sliced, chemically modified tissue that cannot be used for subsequent approaches, such as DNA analysis. Thus, whenever available, type specimens are best candidates to be studied through non-destructive techniques, such as X-ray micro-computed tomography (μCT). Here we present μCT scans of a paratype of the recently described species of land planarian *Obama otavioi* Carbayo, 2016 [4] along with virtual histological sections of the holotype and another paratype of the species. The whole type-material on which description of the species was based consists of only these three specimens.

### Studied specimens

Three specimens comprise the type series. They were collected in Reserva Biológica do Alto da Serra de Paranapiacaba, Santo André, State of São Paulo, Brazil (-23.77697, -46.31212), on 22 September 2012. Photographs of the living animals were taken in the field and in the laboratory with Sony DSC-W125, Sony DSC-W310 and Canon EOS Rebel T5i digital cameras and are freely available at [5]. They were fixed in 10 % formalin and preserved in 80 % ethanol, with the exception of a small piece of tissue that before fixation was cut off and frozen in absolute ethanol.

### Data acquisition and processing

#### Micro-computed tomography procedure

**Fig. 1:**
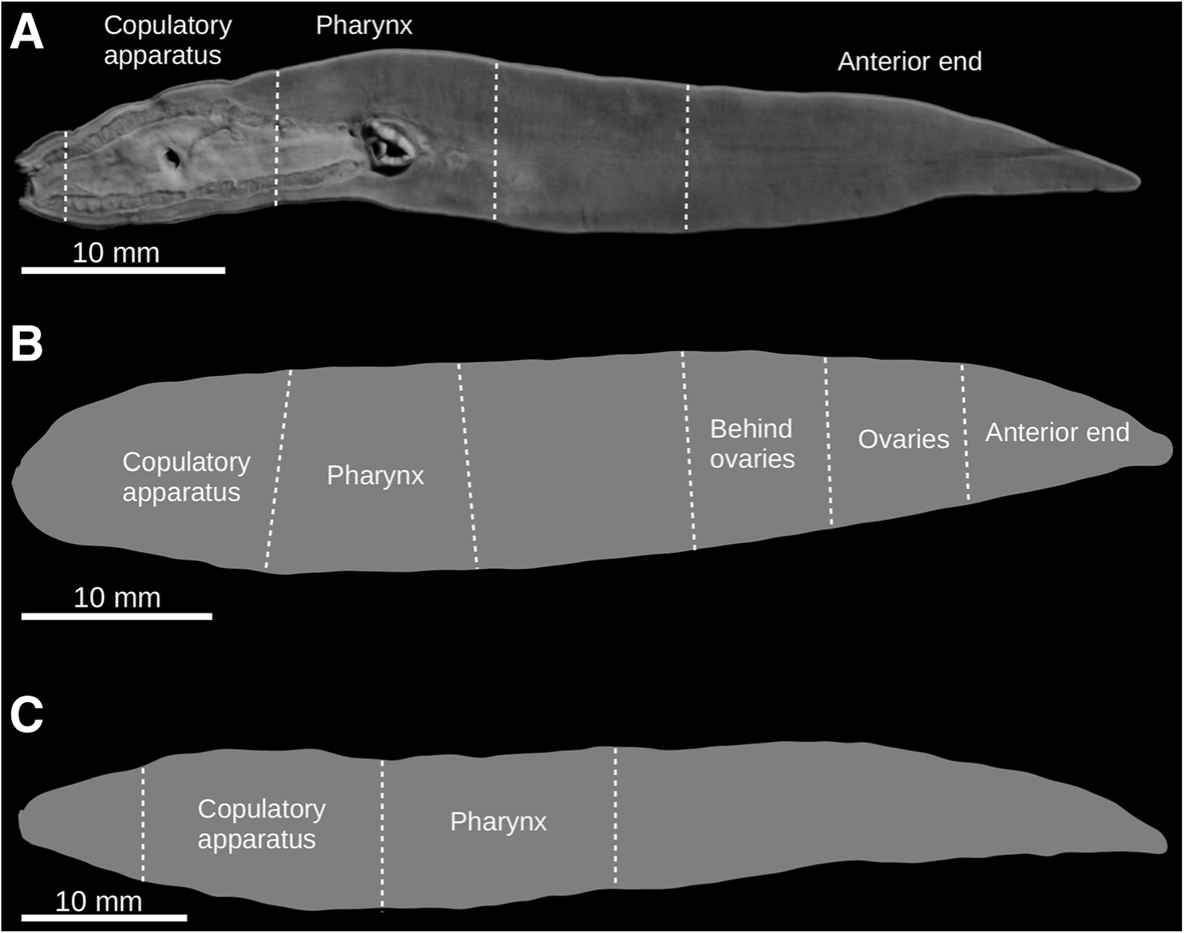
Type-specimens of *Obama otavioi* studied. **a** Ventral view of a 3D rendering of the μCT dataset of paratype MCZ 59100. Dashed lines indicate portions of the body scanned separately: anterior end, pharynx and copulatory apparatus. **b** Outline of holotype with indication of portions of the body that were histologically sectioned. **c** Outline of paratype MZUSP PL 1573 and portions of the body that were histologically sectioned

The paratype MCZ 59100 was submerged in a solution of 0.3 % phosphotungstic acid (PTA) and 3 % dimethyl sulfoxide (DMSO) in 95 % hydrated ethanol, following the protocol by Fernández et al. [6] for earthworms. The time of immersion in this solution was extended to 90 days. The specimen was then rinsed in 95 % ethanol and placed into a plastic straw with 95 % ethanol for preliminary scans. Scans were performed with the X-ray μCT SkyScan 1173 scanner (Bruker MicroCT, Kontich, Belgium) equipped with a Hamamatsu 130/300 tungsten X-ray source and a Flat Panel Sensor camera detector with 2,240 × 2,240 pixels. To enhance the contrast, the specimen was restained with 1 % iodine in absolute ethanol (see detailed description in Carbayo et al. [4]) and new scans were performed. The final datasets obtained consist of four scans (Table 1, Fig. 1a), one at low resolution of the entire body and three scans, at higher resolution, of the anterior tip of the body, the pharynx and the copulatory apparatus, respectively. The scan of the entire specimen at low resolution elucidated the organization and distribution of internal organs, whereas the remaining scans, taken at higher resolution, were aimed at viewing minute structures. Scanning parameters for the four scans are summarized in Table 1; additional parameters can be found in the log file (.log) of each dataset folder available at GigaDB [7]. Each scan resulted in a set of projection images in tagged image file format (TIFF, .tif). No binning protocols were used during data acquisition. The projection images covered 2,240 × 2,240 pixels at 16-bit dynamic range. Reconstruction of the two-dimensional (2D) projection images into a three-dimensional (3D) volumetric image stack was performed using the software NRecon 1.6.6.0 (Bruker microCT, Kontich, Belgium). This program runs under the reconstruction engine NReconServer 1.6.6. The output format for the 3D volumetric image stacks was bitmap image file (BMP,.bmp) at 8-bit dynamic range and 2,240 × 2,240 pixel size, but they can also be reconstructed as a TIFF. In order to reduce final file size, the volume of interest) function, a 3D cropping tool, was used to remove uninformative parts of the data following reconstruction. This resulted in changes to the overall pixel dimensions of each reconstructed image stack but did not lead to spatial distortions in any of the three dimensions.

**Table 1:** Overview of the paratype MCZ 59100 mCT dataset deposited in GigaDB

Region of the body	Entire body (except tail)	Anterior end	Pharynx	Copulatory apparatus
Image pixel size	9.95	6.04	6.04	6.04
Source voltage (kV)	35	34	35	35
Source current (μA)	170	160	160	190
Exposure (msec)	1050	1200	1200	1200
Degree rotation step	0.16	0.07	0.07	0.1
Frame averaging	6	9	12	14
Connected scans	5	3	1	1
Projection files	7505 x.tif, 5 x.log	7202 x.tif, 3 x.log	2401 x.tif, 1 x.log	2401x.tif, 1 x.log
Name of projection files	28-F5434-inteiro19abril_#.tif	27-F5434-PA_#.tif	28-F5434-F_18abril_#.tif	F5434-otavioi-AC_VI_#.tif
Total size of projection files (GB)	64	72.3	24.1	24.1
Reconstruction files	7241 x.bmp 1 x.log	4353 x.bmp, 1 x.log	1903 x.bmp, 1 x.log	1832 x.bmp, 1 x.log, 1 x.db, 2 x.tif
Name of reconstruction files	28-F5434-inteiro19abril__rec#.bmp	27-F5434-PA__rec#.bmp	28-F5434-F_18abril__rec#.bmp	F5434-otavioi-AC_VI__rec#.bmp
Total size of reconstruction files (GB)	7	5.5	4.7	4.7

See also Fig. 1a

#### Histological slide digitization

The holotype and paratype MZUSP PL 1573 were histologically processed, sectioned and stained using the Mallory method as modified by Cason [8]. A total of 166 histological slides were obtained from the holotype (Fig. 1b), and 107 from paratype MZUSP PL 1573, as follows: Holotype MZUSP PL 1574 (field number, F5470): anterior end: transverse sections on 40 slides; anterior region 2: sagittal sections on 35 slides; anterior region 3: horizontal sections on 24 slides; pharynx: sagittal sections on 28 slides; copulatory apparatus: sagittal sections on 49 slides. Paratype MZUSP PL 1573 (field number, F5435): pharynx: sagittal sections on 46 slides; copulatory apparatus: sagittal sections on 61 slides (Fig. 1c).

A total of 81 histological slides were digitized with slide scanner Zeiss Axio Scan Z1 (Table 2). For each slide, a preview was taken for automatic tissue detection. These selections were then refined manually. To obtain high-resolution images, tissue sections were subdivided into multiple small images, or tiles, that were photographed with bright field imaging mode with a 40× objective. Focus was applied every fourth or sixth tile. Z-stacking was activated to obtain a single sharp plane image; this is valuable when sections on the histological slides are uneven. ZEN software (Carl Zeiss Microscopy) automatically produced aligned tiles as a single virtual histological slide in.czi format, which are freely available at GigaDB [7].

**Table 2:** Overview of the virtual slides of the holotype and paratype MZUSP PL 1173 dataset deposited in GigaDB

Specimen	Holotype					Paratype MZUSP PL 1573	
Region of the body	Anterior end	Ovaries	Behind ovaries	Pharynx	Copulatory apparatus	Pharynx	Copulatory apparatus
Name of file folder	09-Holotype(MZUSPPL1574)-histology-AntEnd	10-Holotype(MZUSPPL1574)-histology-Ovaries	11-Holotype(MZUSPPL1574)-histology-behindOvaries	12-Holotype(MZUSPPL1574)-histology-Pharynx	13-Holotype-Histology-CopApp	14-Paratype-MZUSP-PL1573(F5435)-histology-Pharynx	15-Paratype-MZUSP-PL1573(F5435)-histology-Copulatory
Plane of section	Transverse	Sagittal	Horizontal	Sagittal	Sagittal	Sagittal	Sagittal
Slides digitized (of total)	10 (40)	9 (35)	11 (24)	8 (28)	15 (49)	10 (46)	18 (61)
Total size of scans (GB)	6.96	28.2	47.6	32.5	73.7	44.2	57.7

See also Fig. 1b,c

### Data quality

#### μCT-based images

Highest contrast and sharpness were achieved at about 34–35 kV, 160–190 μA and 1,000–1,200 ms exposure. The highest voxel resolution achieved with the scan was 6.04 μm. High quality scans of the entire animal were avoided as to not create large file sizes. Instead, each individual was scanned at lower resolution and small sections were scanned at high resolution to generate more manageable file sizes.

Eyes and the prostatic vesicle are small structures that are less easily outlined in the scans. Eyes were partially masked by body pigmented cells. Despite its relatively large size, the vesicle stained weakly so it was laborious to outline it. Structures less than 5 μm in size, such as sensory pits and single muscle cells, were also nearly indistinguishable on the μCT-based images.

#### Virtual histological slides

Almost all images of virtual histological slides obtained were of high quality. A few images present blurry portions or border tiles were visible. This might be due to tiny debris remaining on the cover slides and to technical failure at digitization, respectively. Given that these regions occurred very scattered along the serial sections, they do not hinder image analyses aimed at taxonomic investigation.

### Potential uses

Freely available digital image datasets are suitable for study of museum specimens across the globe, without the potentially prohibitive costs of travel or museum loans. These cybertypes [9] can be non-destructively studied in ways that would otherwise damage tissues or prevent DNA extraction. The non-destructive nature of μCT allows for the study of type specimens, and for molecular work after scanning. Furthermore, μCT lends itself to rapid mass scanning of taxonomic groups. This method of visualization could then be implemented to distinguish minute morphological characters and possibly identify new structures for taxonomic division among cryptic species. Virtual slides allow verification of manual 3D reconstructions, which in part depend on individual interpretation of the serial sections (see example in [10]). They contain much more information than what is described in taxonomic approaches, so they also allow the exploration of new unstudied morphological characters.

## Discussion

Although these animals are relatively large, some of the taxonomically relevant structures, such as sensory pits and muscle organization, require high resolution of μCT-based images to be detected. Consequently, high-performance computers with suitable memory RAM and hard disk space are needed, and a rapid internet connection is essential. With these technological requirements, μCT-based imagery and virtual histological slides will be the best way to surmount the hindrance of specimen loans. μCT is especially relevant for getting access non-destructively to morphology of rare or unique specimens, such as type specimens. Digitization of histological slides of type specimens is the best way to facilitate study of this material without the inherent risk of damage when transporting loans.

## Availability and requirements

### Data availability

The datasets are available at GigaDB [7]. They consist of four folders containing μCT-based images plus seven folders with virtual histological slides. Dataset name: μCT scans and digitized histological slides of type series of the land planarian *Obama otavioi*.

### Data requirements

Downloaded reconstructed μCT-based images can be visualized using the ‘File:Import: Image Sequence’ command chain in the Java-based imaging software ImageJ [11]. There is also other 2D and 3D free visualization software [12]. Datasets can also be opened and manipulated using the freely available software from SkyScan in both 2D and 3D formats [13]. For μCT-based images a computer system with over about 24 GB random access memory (RAM) should be used.

Downloaded virtual histological sections can be visualized using Bio-Formats Importer plugin installed in the Fiji distribution of ImageJ [14]. They also can be visualized with ZEN. For virtual slides a computer system with over about 4 GB main RAM should be used.

## Abbreviations

μCT: micro-computed tomography
